# Factors Influencing Salt-Reducing Behavior in Young Adults: a Pilot Cross-Sectional Study from Kazakhstan

**DOI:** 10.5195/cajgh.2020.415

**Published:** 2020-03-31

**Authors:** Mina Aubakirova, Marat Sultanov, Aidarkhan Izimov, Yesbolat Sakko, Torekhan Bex, Anuar Mussagazin, Raushan Alibekova

**Affiliations:** 1 School of Medicine, Nazarbeyev University, Nur-Sultan, Kazakhstan

**Keywords:** Attitudes, Behaviors, Kazakhstan, Knowledge, Salt

## Abstract

**Introduction:**

In Kazakhstan, a post-Soviet country in Central Asia, salt intake is estimated as high, potentially contributing to the morbidity and mortality from cardiovascular diseases. The aim of this study was to explore salt intake in residents of the capital of Kazakhstan, Nur-Sultan.

**Methods:**

An online cross-sectional survey of knowledge, attitudes, and behaviors on salt intake among young adult residents of the capital city of Kazakhstan was conducted (*n* = 237). Bivariate and multivariate linear regression analyses were performed.

**Results:**

Although 95% (n=225) reported knowledge on the adverse health effects of high salt intake, older respondents were more aware of its association with high blood pressure (p = 0.007), heart disease (p = 0.037), and heart attack (p = 0.002). Only one-third (n=79) correctly identified the recommended level of daily salt intake. Females reported more awareness of Kazakhstani people consuming salt more than recommended (p = 0.0027) and that processed products constituted the major source of salt in diet (p = 0.007). General dietary concern (p < 0.001), high self-assessmen of salt intake (p < 0.001), and older age (p = 0.012) were found to be adjusted predictors of salt-reducing behavior.

**Conclusions:**

Lack of reported knowledge on salt-health relationship is of concern, especially among young males. A greater dietary concern and individual awareness of the excessive salt consumption is likely to assist in reducing salt intake. Further studies are required to validate the findings of this pilot study on a bigger population level in order to provide a basis for future salt related interventions and policy changes in Kazakhstan.

Central Asia has been reported as one of the regions with the highest burden of cardiovascular diseases (CVD) in the world^1^. In Kazakhstan, incidence of CVDs increased from 8,600 cases per 100,000 in 2005 to 15,500 cases per 100,000 in 2016^2^, ^3^.

Diet has been identified as one of the preventive measures for decreasing risk of chronic illnesses along with exercising and avoiding smoking and consumin alcohol^4^. Among dietary practices, excessive salt intake is commonly recognized as the factor associated with cardiovascular diseases^5^^–^^7^. Excessive salt intake has been determined to be a risk factor for high blood pressure, and a related global target of 30% reduction has been included in the Global Action Plan for the Prevention and Control of Noncommunicable Diseases for 2013-2020^8^.

The World Health Organization (WHO) advises the daily intake of salt not to exceed five grams^9^. Multiple studies report that this threshold is crossed in numerous regions,^10^, ^11^ including Central Asia region^12^. According to the WHO, in Kazakhstan, the daily intake of salt surpasses the WHO recommended limit by nearly fourfold^13^.

Studies vary with regard to how awareness of the negative impact of salt on health and attitudes toward salt consumption in general influence dietary behaviors. Some have found that the higher the knowledge, the more conscientious food choices are^7^, while other studies have observed absence of willingness of people to change their eating habits, even when they realize the negative side-effects of high amounts of salt consumption^14^.

Given the high incidence of CVDs and the higher than recommended levels of salt consumption among the Kazakhstani population, we have conducted a survey of knowledge, attitudes, and behaviors (KABs) related to salt intake among young residents of the city of Nur-Sultan. The city was chosen because it is the capital, so it attracts, and therefore represents, residents from all over the country.

## Methods

### Study design and participants

Since knowledge, attitudes, and behaviors are more easily altered in young people, the study population of the research was chosen to be young people aged 18 and above residing in Nur-Sultan. The younger generation has widespread access to the Internet. According to the Department of Statistics of the Republic of Kazakhstan, in 2018, 90.1% of Nur-Sultan population in the 16–44 age group had access to the Internet, while among all Internet users of Nur-Sultan, 13.4% and 67.9% fell in the 16–24 and 25–64 age groups, respectively^15^. Lack of a common sampling frame for mobile phone users, as well as the unpopularity of landline phones among the target group, made random digit dialing an unfeasible means of data collection. Therefore, an Internet survey was decided to be conducted.

### Data collection

A survey was devised and pilot tested on 20 participants. After collecting feedback from the respondents, appropriate amendments were implemented, and the final survey was launched using Qualtrics platform, where it was accessible during 15 - 30 March 2018. Snowball sampling was used to recruit participants through ads on social network pages relevant to the target demographic, outlining the survey aims and offering interested young adults to participate. Such pages included those related to local universities and leisure activities in social media, namely Vkontakte and WhatsApp. Since no individual invitations were sent, estimating the response rate was not possible. A small number of responses were excluded from the final sample due to completion of only the initial questions related to demographic information.

Before proceeding to the questionnaire, the participants were presented with information on the survey's purposes and their right to withdraw from completing the survey at any point; therefore, informed consent was assumed for all participants that completed the survey. It also stated that by filling out the survey, they confirmed that they were over the age of 18. No personal identifiable information was collected from the participants. The respondents could choose to complete the questions either in Russian or Kazakh language. No requirement was imposed on the participants to complete all the questions. Ethical approval for the study was obtained from Nazarbayev University Institutional Research Ethics Committee.

### Questionnaire

A questionnaire containing 18 questions was adapted from a questionnaire of KABs related to dietary salt used in the study by Grimes et al^16^. Some alterations to the original survey instrument were introduced. Then, the survey was translated into Russian and Kazakh by two independent translators, and discrepancies in the translations were further assessed by a third translator.

The demographic questions included gender, age, region of origin, education level, and level of involvement in cooking. Age was categorized into four groups (18–20, 21–23, 24–26, and 27–30), which was aimed at facilitating usage of mobile devices. The two questions related to country of birth and language spoken at home were substituted with a question on the region of origin within the country to potentially compare regional dietary differences. The options provided in the question related to education level, which apart from directly reflecting level of education also serves as a proxy to socio-economic status, were redesigned to represent the local education system's specifics.

Several questions similar to the ones in the original survey instrument were used to assess the knowledge related to dietary salt intake. The questions were concerned with the knowledge on: (a) the relationship between sodium and salt, (b) the recommendations related to salt intake, (c) the level of salt consumption in Kazakhstan (in comparison to recommendations), (d) the main source(s) of salt in the diet of Kazakhstan's population, (e) health risks associated with high salt intake, and (f) association of high salt intake with several health outcomes. Categorical response options were provided for each question. For the purposes of multivariate analysis, a knowledge score variable was constructed. A score of 1 was assigned for each correct answer, while 0 was given for wrong responses and if the respondent chose the ‘I don't know’ option. Thus, the knowledge score ranged from 0 to 9.

The correct responses for the question related to the level of salt consumption in Kazakhstan were assumed to be ‘More than needed’ and ‘Too much’, since sodium intake for Kazakhstan and the Central Asia region as a whole has been ranked among the highest in the world^12^, ^13^. For the question related to the main sources of salt in the diet, the correct response was assumed to be ‘Processed foods’, which is consistent with a WHO report from Kazakhstan^13^.

Two questions assessed the participants’ attitudes regarding salt intake. A block question was used to assess the participants’ level of concern for several food-related issues (healthy eating and sugar/salt/fat/saturated fat/calorie content). Scores were assigned for each answer on a Likert type scale with 1 to 5 assigned to answers from ‘Not at all concerned’ to ‘Very concerned’. The concern score was created by combining response scores and used in the multivariate model.

The other question in this section was related to the participants’ own assessment of their salt intake levels in view of the recommendations. If a respondent indicated that their personal consumption of salt was higher than the recommended level, then the response was assigned a score of 1, with all the other response categories given a score of 0. This variable was used in the regression analysis as a binary predictor variable.

These questions were concerned with the participants’ past and current behaviors regarding salt intake. Three questions were designed with a 5-point frequency scale from ‘Never’ to ‘Always’, asking participants about their habits of (a) adding salt during cooking, (b) adding salt while eating, and (c) placing a saltshaker during meals. Scores were assigned from 1 to 5, with 1 representing the lowest salt-reduction behavior (e.g. if the respondent reported always adding salt while cooking).

The next four questions assessed several behaviors related to salt reduction strategies that the participants may have employed in the past month on a 5-point frequency scale from ‘Never’ to ‘Always’. If a person reported to have never engaged in a particular behavior related to salt reduction, the response was assigned a score of 1, while the highest score was given for the highest salt-reducing behavior.

All the scores from 1 to 5 for the seven questions on salt-reduction behavior were combined to form a behavior score ranging from 7 to 35 for the multivariate model.

### Data analysis

The data collected through the Qualtrics survey system^17^ were exported into spreadsheet format. Chi-square test of independence and Fisher's exact test were used for bivariate analyses. Multivariate linear regression models were constructed with salt reduction behavior score as the outcome and knowledge score as the primary predictor, adjusted for dietary concern score, perception of personal salt consumption, gender, age, and region. Statistical analysis was performed using Stata software version 14.2^18^. Statistical significance was reported at α = 0.05.

## Results

### Knowledge related to salt intake

The majority of the respondents (62.87%, n=149) correctly indicated that salt contains sodium (Table 2). Females were better informed than male respondents that Kazakhstani people consumed salt more than recommended (p = 0.0027) and that processed meat and other processed products constituted the major sources of salt in diet (p = 0.007) (Supplementary Table 1). Almost 95% (n=225) of the participants replied that eating too much salt could damage health. However, older age groups were more aware than the younger age groups of the association between excessive salt consumption and high blood pressure (p = 0.007), heart disease (p = 0.037), and heart attack (p = 0.002). Similarly, respondents with general secondary education had lower awareness than those with higher levels of education of the association of excess in salt with heart disease (p = 0.007) and heart attack (p=0.013).

**Table 1.** Socio-demographic characteristics of participants (n=237)

Characteristic	N	%
Gender		
Male	84	35.44
Female	153	64.56
Age group		
18–20	78	32.91
21–23	42	17.72
24–26	74	54.02
27–30	43	18.14
Region of origin		
Nur-Sultan	46	19.41
Almaty	25	10.55
Central Kazakhstan	28	11.81
East Kazakhstan	14	5.91
North Kazakhstan	50	21.09
South Kazakhstan	44	18.57
West Kazakhstan	30	12.66
Language of survey completion		
Kazakh	11	4.6
Russian	226	95.4
Level of education		
Higher	188	79.32
Professional secondary	10	4.22
General secondary	39	16.46
Level of involvement in cooking		
Always	46	19.41
Often	76	32.07
Sometimes	57	24.05
Rarely	48	20.25
Never	10	4.22

### Attitudes related to salt intake

**Figure 1.**
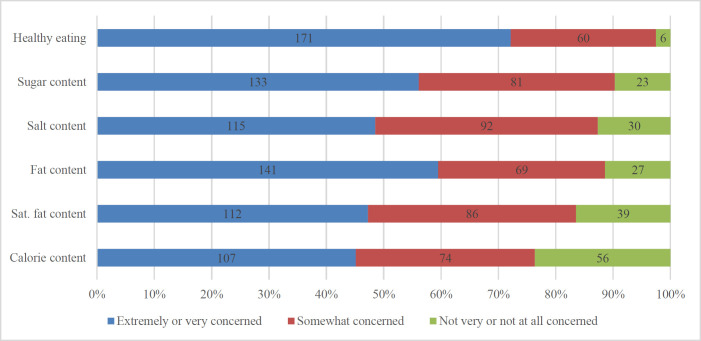
Level of concern for food-related issues

Roughly half of the participants were concerned about the salt content in their diet (Figure 1). Women, however, showed higher concern regarding salt content in food compared to men, although this finding was not significant (p = 0.064). With regard to concern on other dietary contents, the respondents with general secondary education had lower levels of concern about the amount of fat in food (p = 0.013), whereas female respondents were concerned with the amount of saturated fat in products more than males (p = 0.018) (Supplementary Table 2).

**Table 2.** Knowledge & attitudes related to salt intake

Question	N	%
Salt may be defined in the product's contents as ‘salt’ and as ‘sodium’.		
What is the relationship between salt and sodium?		
They are exactly the same	39	16.46
**Salt contains sodium**	149	62.87
Sodium contains salt	9	3.80
Don't know	40	16.88
Health professionals recommend that we should eat no more than a certain amount of salt each day. How much salt do you think it is?		
3 grams	94	39.66
**5 grams**	79	33.33
8 grams	17	7.17
10 grams	13	5.49
15 grams	5	2.11
Don't know	29	12.24
Do you think eating too much salt could damage your health?		
**Yes**	225	94.94
No	3	1.27
Don't know	9	3.8
Which, if any, of the following do you think is linked to eating too much salt?		
High blood pressure		
**Yes**	145	61.18
No	20	8.44
Don't know	72	30.38
Kidney disease		
**Yes**	198	83.54
No	9	3.80
Don't know	30	12.66
Heart disease		
**Yes**	139	58.65
No	27	11.39
Don't know	71	29.96
Heart attack		
**Yes**	108	51.05
No	30	13.08
Don't know	83	35.86
In your opinion, how much salt do Kazakhstani people consume?		
**Too much**	19	8.02
**More than needed**	146	61.6
Normal amount	62	26.16
Less than needed	2	0.84
Don't know	8	3.38
Which of the following do you think is the main source of salt in the diet of Kazakhstan's population?		
Salt added during cooking or at the table	101	42.62
**Salt contained in processed meat products and in other processed products**	124	52.32
Salt contained in natural food products	4	1.69
Don't know	8	3.38
How do you think your daily salt intake compares to the amount of salt recommended by health professionals?		
Less than recommended	25	10.55
About the right amount	124	52.32
More than recommended	69	29.11
Don't know	19	8.02

^*^ Correct responses for knowledge questions are in bold

### Behaviors related to salt intake

**Figure 2.**
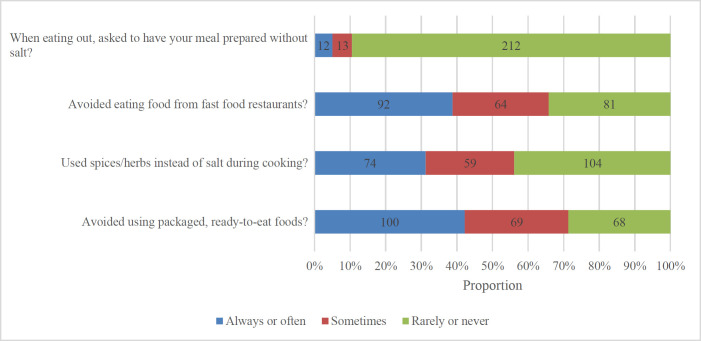
Behavioral practices to reduce salt intake performed in the past month

More than 90% of the sample reported never or rarely asking to have a meal prepared without salt when eating out (Figure 2). Among the respondents, the oldest age group (27–30 years old) was more likely than the other age groups to avoid eating at fast food restaurants as a salt-reducing practice (p = 0.002) (Supplementary Table 3). Respondents with higher and general secondary education levels were more likely to avoid adding salt to food during meals than those with professional secondary education (p = 0.007).

**Table 3.** Multivariate model: predictors of salt-reducing behavior

Predictor variables		Coefficient	95% CI	P
Knowledge score		0.263	−0.021; 0.547	0.069
Concern score		0.269	0.142; 0.396	**<0.001**
Assessment of own level of salt consumption compared to recommendations^*^		−3.023	−4.211; −1.834	**<0.001**
Gender^**^		−0.518	−0.169; 0.653	0.385
Age^***^	21–23	0.759	−0.873; 2.392	0.361
	24–26	0.535	−0.808; 1.878	0.433
	27–30	2.079	0.452; 3.705	**0.012**
Region of origin^****^	Central Kazakhstan	0.772	−1.066; 2.610	0.409
	West Kazakhstan	0.409	−1.329; 2.146	0.644
	South Kazakhstan	1.273	−0.054; 2.600	0.060
	East Kazakhstan	0.193	−2.229; 2.614	0.876

* Binary variable with all responses except for ‘More than recommended’ combined as reference group

** Females as reference group

*** 18–20 as reference group

**** North Kazakhstan as reference group

### Multivariate analysis

Level of knowledge was not associated with salt-reducing behavior at p = 0.069, adjusting for age, gender, region of birth, and level of dietary concern variables. On the other hand, dietary concern score was found highly associated with salt reduction behavior at p < 0.001, as was the variable of self-assessment of salt intake. Among demographic predictors, a statistically significant difference in salt reducing behavior was observed between the 18–20 and 27–30 age groups (p = 0.012). Neither gender nor region of origin was significantly associated with the person's salt-related behavior.

## Discussion

The main findings of the study suggest that level of knowledge is not a significant predictor of salt-reducing behavior, adjusting for age, gender, region of birth, and level of dietary concern variables. Young adults who were concerned in general about their diet and those who self-assessed their salt intake level as high reported salt-reducing behaviors more frequently. Participants aged 27–30 reported higher engagement in salt-reducing behavior than those aged 18–20.

The local relevance of the research topic is set to increase in the coming years as the burden of cardiovascular diseases continues to exacerbate, thereby increasing the demand for preventive population-wide interventions.

Although an overwhelming majority were aware of the increased health risk as a result of high salt intake, which is consistent with similar studies conducted in other cities of Kazakhstan^13^ and internationally^16^, ^19^, younger participants were less aware of the association of salt with specific cardio-vascular health outcomes. Educating on these relationships could be the purpose of salt-related local interventions, especially given the morbidity and mortality rates associated with CVD in Kazakhstan, and should specifically target younger populations.

Yet the effectiveness of purely educational interventions may be debatable. For example, a KABs study in Australia revealed that despite a decent level of awareness of adverse health impacts of excess salt consumption, the respondents were not willing to reduce these consumption amounts due to abundant promotion of inexpensive products high in salt and the lack of proper food labelling^6^. Therefore, public health programs should target these aspects apart from focusing on awareness-raising.

In the current study, the main perceived sources of salt (added during cooking or at the table and salt contained in processed products) were also among the identified leading sources of salt in a study of salt intake in Turkey^20^. Again, awareness of the main sources of salt is not sufficient for encouraging salt-decreasing behaviors and an emphasis on practical skills may be necessary^19^. Thus, specific interventions targeting cooking practices may be useful. In Kazakhstan, such practical interventions should target women, who are better informed about the primary sources of salt in food and are usually the primary cooks in traditional Kazakh families.

Among the respondents, those generally concerned with food contents reported higher engagement in salt-decreasing behaviors. Specifically, women were more concerned with the amount of saturated fat in food; therefore, interventions on salt-reduction practices for women could be integrated into saturated-fat reduction or into general healthy diet interventions. Higher self-assessed salt intake level in our study was associated with frequent salt-reducing behavior, similarly to the findings of a previous study from Australia^16^. A recent report on a WHO study of salt-related KABs in two regions of Kazakhstan did not examine specifically the association between attitudes and behaviors; however, it claims that only 10% of the respondents evaluate their consumption of salt as excessive^13^.

Salt-reduction behaviors were higher in the oldest participants than in the younger ones. These findings are similar to results of a cohort study by Vega-Vega et al. (2018), measuring sodium in urine and through dietary recall, which also observed associations of age (p = 0.03) and male gender (p < 0.001) with sodium intake, adjusting for intake of calories, median urinary iodine excretion, and body-mass index^21^. A study from two Kazakhstani regions also shows that males have higher sodium concentration in urine than females^13^.

The study is subject to coverage bias due to the selected data collection mode. However, our target population of young adults are predominantly active Internet users; moreover, the anonymous online data collection may have reduced the possibility of social desirability bias. The results have limited generalizability because of the sampling technique and given that the majority of the respondents had higher education, younger age, and urban status. Furthermore, there is a possibility of answering the questionnaire twice or multiple times in the Internet survey. Finally, due to the cross-sectional design study, results need to be interpreted with caution.

Future research could use other methodology to address the limitations of the current paper and add to the validity of the present results. Also, further studies will be required to evaluate the effectiveness of salt-related interventions.
